# Immunity‐targeted approaches to the management of chronic and recurrent upper respiratory tract disorders in children

**DOI:** 10.1111/coa.13335

**Published:** 2019-04-14

**Authors:** Wojciech Feleszko, Ricardo Marengo, Antonio Sousa Vieira, Karol Ratajczak, José Luis Mayorga Butrón

**Affiliations:** ^1^ Department of Paediatric Respiratory Diseases and Allergy The Medical University of Warsaw Warsaw Poland; ^2^ Department of Otorhinolaryngology and Audiology CEMIC Institute Buenos Aires Argentina; ^3^ Department of Otorhinolaryngology Lusíadas Hospital Porto Portugal; ^4^ Department of Otorhinolaryngology National Institute of Pediatrics Cuicuilco Mexico; ^5^ Master of Science Program Postgraduate Unit Faculty of Medicine National University of Mexico Cuicuilco Mexico

**Keywords:** children, chronic, immunity‐targeted, recurrent, respiratory tract infections

## Abstract

**Background:**

Upper respiratory tract infections (URTIs), including rhinitis, nasopharyngitis, tonsillitis and otitis media (OM), comprise of 88% of total respiratory infections, especially in children. Therefore effective prevention and treatment of RTIs remain a high priority worldwide. Preclinical and clinical data highlight the rationale for the use and effectiveness of immunity‐targeted approaches, including targeted immunisations and non‐specific immunomodulation in the prevention and management of recurrent upper RTIs.

**Objective of review:**

The idea of this review was to summarise the current evidence and address key questions concerning the use of conservative and immunity‐targeted approaches to recurrent and chronic URTIs, with a focus on the paediatric population.

**Search strategy/Evaluation method:**

Literature searches were conducted in March 2017 and updated in September 2017 using: Academic Search Complete; CENTRAL; Health Source: Nursing/Academic Edition; MEDLINE; clinicaltrials.gov; and Cochrane databases. In total, 84 articles were retrieved and reviewed. Two independent researchers focused on primary and secondary endpoints in systematic reviews, meta‐analyses and randomised, controlled trials, using immunity‐directed strategies as the control group or within a subpopulation of larger studies. Existing guidelines and interventional/observational studies on novel applications were also included.

**Results:**

Children are particularly susceptible to RTIs due to the relative immaturity of their immune systems, as well as other potential predisposing factors such as day care attendance and/or toxic environmental factors (eg increased pathogenic microbial exposure and air pollutants). Recurrent URTIs can affect otherwise healthy children, leading to clinical sequelae and complications, including the development of chronic conditions or the need for surgery. Available pre‐clinical and clinical data highlight the rationale for the use and effectiveness of immunity‐targeted approaches, including targeted immunisations (flu and pneumococcal vaccines) and non‐specific immunomodulation (bacterial lysates), in the prevention and management of recurrent croup, tonsillitis, otitis media, recurrent acute rhinosinusitis and chronic rhinosinusitis.

**Conclusions:**

In this review, we summarise the current evidence and provide data demonstrating that some immunity‐targeted strategies, including vaccination and immunomodulation, have proved effective in the treatment and prevention of recurrent and chronic URTIs in children.

**Keypoints**

- Upper RTIs comprise 88% of total respiratory infections
- Children are particularly susceptible to RTIs because their immune system has yet to fully mature
- Recurrent URTIs can affect otherwise healthy children, leading to clinical sequelae and complications
- Owing to the high morbidity, mortality and healthcare costs, effective prevention and treatment of RTIs are a high priority worldwide
- Pre‐clinical and clinical data highlight the rationale for the use and effectiveness of immunity‐targeted approaches, including targeted immunisations and non‐specific immunomodulation in the prevention and management of recurrent upper RTIs.

## 1 RESPIRATORY TRACT INFECTIONS

### 1.1 Introduction

Upper respiratory tract infections (URTIs; also known as ENT infections), including rhinitis, nasopharyngitis, tonsillitis and otitis media (OM), comprise 88% of total respiratory infections.^1^ The aetiology of URTIs is mostly viral, being primarily caused by rhinovirus (HRV), parainfluenza, respiratory syncytial virus (RSV), influenza, adenovirus and coronavirus. Upper respiratory tract infections are more common in the autumn/winter in Europe and North America, and during the rainy season in tropical countries.^2^

Children are prone to developing RTIs because their immune system has yet to fully mature.^3^, ^4^, ^5^ Increased exposure to viral infections during day care attendance, as well as other social and environmental factors, can increase the risk of RTI.^4^ The respiratory system is a primary target for key air pollutants, which can increase the risk of acute and recurrent RTIs.^6^ Most of these pollutants disrupt local mucosal innate immunity mechanisms, leading to bacterial colonisation, impaired killing and increased allergen permeability.

### 1.2 Burden and management of RTIs

URTIs are associated with sequelae and complications, including severe lower RTIs (LRTIs), the development of chronic conditions or the need for surgery when recurrent. Owing to the high morbidity, mortality and healthcare costs, effective prevention and treatment of RTIs are a high priority worldwide.^7^, ^8^ Treatment is focused on symptom relief, such as antihistamines and decongestants for nasal congestion^9^ and antitussives for cough.^10^, ^11^ Although antibiotics are only indicated in a minority of patients, they are often unnecessarily prescribed for viral infections against which they have no effect. A recent US report highlighted that ~30% of outpatient, oral antibiotic prescriptions were inappropriately written.^12^ Antibiotic misuse has led to the emergence of resistant bacteria, meaning that higher doses and more advanced generations of drugs are required, and at present, some infected patients cannot be treated adequately.^13^

Owing to the current unmet need for effective, alternative conservative therapies, efforts are being refocused towards preventative strategies including^5^ behavioural intervention; avoidance of environmental risk factors such as passive smoking^14^ and highly polluted city areas; vaccination, such as active targeted immunisation; targeted medical intervention; non‐specific immunostimulation or immunomodulation (eg bacterial lysates)^15^, ^16^, ^17^, ^18^; nutrition, including vitamins (eg C or D) and microelements; and regular physical activity (Table 1). Parents of children with recurrent RTIs may also discuss “immune‐stimulating” therapies that they have seen advertised, such as herbal (eg Echinacea) and homoeopathic remedies, and animal‐derived products (eg cod liver oil and thymus extracts).^5^ Owing to significant heterogeneity and a lack of high‐quality clinical evidence supporting many of these therapies,^19^ here we reviewed existing immunity‐targeted therapies available for ENT infections in children, with an emphasis on non‐specific immunomodulation administered together with the standard of care.

**Table 1:** Current preventative and evidence‐based immunity‐targeted approaches in the management of recurrent URTIs

Environmental	Behavioural	Nutritional	Systemic
Protection from noxious environmental factors (passive smoking, traffic‐related pollution)	Improved hand washing Increased physical activity Avoiding crowded communities	Zinc Vitamin D	Immunisation Oral immunostimulators/Immunomodulators, for example bacterial lysates, herbal products, probiotics, other chemical compounds

URTI, upper respiratory tract infection.

### 1.3 Role of immunomodulation in respiratory tract infections

Immunomodulatory strategies treat RTIs by enhancing local and systemic immune responses. Systemic effects relate to the use of microbial‐derived preparations (bacterial lysates, bacterial ribosomes) that display effects such as increased systemic polyclonal immunoglobulin (Ig) synthesis (both IgA and IgG classes) and activation of several populations of immunocompetent cells (including CD4+ lymphocytes, natural killer cells, and B lymphocytes).^20^ These effects have consequently been reported in both in vitro and in vivo experiments (for review, see Kearney et al).^21^

A number of clinical studies have shown that bacterial lysates and bacterial organelles (eg ribosomes), when applied orally, are effective in preventing both URTI and LRTIs in children, decreasing the number, duration and severity of infectious episodes, reducing antibacterial use and decreasing the number of work/school absences.^22^, ^23^ Immunomodulation with bacterial lysates is of particular interest because several randomised, controlled trials, summarised in systematic reviews or meta‐analyses, have demonstrated their efficacy in children with RTIs.^19^, ^24^, ^25^ A plausible mechanism of action for bacterial lysates has been proposed, which is not only based on an increase in non‐specific immunoglobulin A response against pathogens at mucosal surfaces, but also on activation of mucosal dendritic cells by pattern recognition receptor‐dependent signalling.^26^ They act on both innate and adaptive immune responses, with recent findings supporting antiviral effects (eg increased interferon [IFN]‐γ, IFN‐α and IFN‐β).^26^, ^27^, ^28^ Interestingly, application of bacterial lysates also produces systemic effects, including increased serum immunoglobulin concentrations^29^ and systemic CD4+ T‐helper cell responses to bacterial antigens. For a comprehensive review of the mechanism of action of bacterial lysates, see Kearney et al 2015 and Esposito et al 2018.^21^, ^30^ Immunomodulation with bacterial lysates has been empirically well accepted and used by the medical community for decades for treating RTIs,^19^ and one of these commercially available compounds (OM‐85) has been incorporated into various clinical treatment guidelines such as those from the Pan American Association of Otorhinolaryngology and Head and Neck Surgery^31^ and a European Position (EPOS) paper^32^ (albeit only for the management of specific chronic respiratory conditions in adults, as studies in children were not comprehensively evaluated) with the highest recommendation grade. More recently, OM‐85 has been recommended for the prevention of RTIs in children in several international guidelines.^33^, ^34^ Systemic strategies include the application of medications or dietary factors that may enhance systemic immune responses, such as zinc preparation intake, vitamin D supplementation and probiotics; however, their clinical effectiveness remains debatable.

In this review, we want to highlight emerging, evidence‐based approaches for conservative management of the most common recurrent and chronic URTIs in children, with an emphasis on the role of immunomodulation.

## 2 METHODOLOGY

### 2.1 Search strategy and evaluation method

Literature searches were initially conducted in March 2017 and then updated in September 2017 using: Academic Search Complete; CENTRAL; Health Source: Nursing/Academic Edition; MEDLINE; clinicaltrials.gov; and Cochrane databases. In total, 84 articles were retrieved and reviewed. Two independent researchers focused on the primary and secondary endpoints reported in systematic reviews, meta‐analyses and randomised, controlled trials, using immunity‐directed strategies as the control group or within a subpopulation of larger studies. Existing guidelines and interventional or observational studies on novel applications were also included in the literature searches.

### 2.2 Ethical considerations

As no human participants were involved in the development of this review, no ethics committee (Institutional Review Board) approval was sought or obtained, and it was not necessary to obtain informed consent from patients.

## 3 CROUP

### 3.1 Introduction

Children with recurrent or severe croup are commonly referred to a paediatric otolaryngologist or ENT specialist for further assessment and exclusion of an underlying anatomical or congenital upper airway disorder.

### 3.2 Epidemiology

Croup occurs at a rate of ~5/100 in the second year of life, with a peak incidence between 6 months and 3 years of age.^35^ Around 15% of annual clinic and emergency department visits for paediatric RTIs are due to croup.^35^ Recurrent croup in children is typically defined as more than three episodes.^36^

### 3.3 Aetiology

Croup is primarily caused by parainfluenza viruses 1, 2 and 3, as well as RSVs. The viruses are generally spread by direct inhalation from a cough or sneeze, or by contamination of the hands following contact with fomites, with subsequent transference to the mucosa of the eyes, nose or mouth.

### 3.4 Treatment of acute or recurrent episodes

The treatment of croup is dependent on the severity of the upper airway obstruction and the risk for rapid deterioration of the patient's condition. Mild‐to‐moderate croup is typically treated with anti‐inflammatory agents (nebulised or oral steroids), while adrenaline is required urgently in cases of severe croup.^37^ The use of systemic corticosteroids early in the disease process reduces hospitalisation rates,^38^ although 6‐10% of patients still require hospitalisation.^39^

### 3.5 Prevention of recurrent episodes and the role of immunomodulation

Several evidence‐based treatment guidelines for croup are currently available online, including those from the UK,^40^ Australia,^41^ Poland^42^ and Finland.^43^ However, these guidelines provide only general measures for lowering the risk of respiratory infections. There are currently no vaccines targeting the viruses responsible for croup, despite a number of attempts to develop them.^44^, ^45^, ^46^ Achieving satisfactory immunogenicity has proved challenging,^47^, ^48^ although ongoing clinical trials (see clinicaltrials.gov: NCT00186927) are assessing new, well‐tolerated vaccines. Since most croup‐causing viruses also cause URTIs, there could be a therapeutic role for other immunomodulatory strategies, although well‐defined, controlled studies are required.

## 4 RECURRENT TONSILLITIS

### 4.1 Epidemiology

Tonsillitis is more common among children than adults.^49^, ^50^ In the USA, sore throat accounts for 2.1% of outpatient visits,^51^ with a prevalence of bacterial tonsillitis of 15‐30% among affected children.^52^, ^53^, ^54^ Recurrent tonsillitis in children is defined as multiple episodes of acute tonsillitis in a year.^55^ The burden of recurrent tonsillitis is substantial and it may lead to peritonsillitis. However, the overall frequency and magnitude of the problem of recurrent tonsillitis remains unclear.

### 4.2 Treatment of acute and recurrent episodes

The treatment of tonsillitis in children focuses on reducing symptoms, avoiding complications, decreasing the number of disease‐related school absences and improving quality of life. According to clinical practice guidelines, the first‐line treatment for bacterial tonsillitis should be a narrow‐spectrum antibiotic (eg penicillin).^16^, ^49^, ^50^ However, some European countries, including Germany, the UK and the Netherlands, only recommend antibiotics in certain high‐risk patients.^56^ The widely accepted indications for tonsillectomy in children are at least seven well‐documented, clinically significant, adequately treated sore throat (defined as “acute pharyngitis, tonsillitis or acute exudative tonsillitis”) in the preceding year, or at least five such episodes in each of the preceding 2 years, or at least three such episodes in each of the preceding 3 years.^49^, ^50^ Surgery should also be considered if the episodes are disabling and prevent normal functioning.

### 4.3 Prevention of recurrent episodes and role of immunomodulation

**Figure 1:**
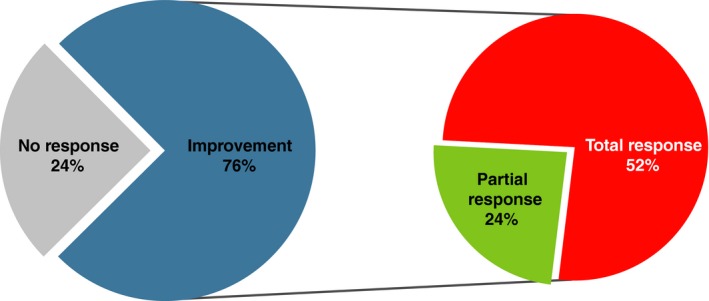
Bacterial‐derived immunomodulator in the prevention of acute tonsillitis (Reprinted from Bitar & Saade, Copyright © 2013, with permission from Elsevier).^57^ “Total response” was defined as a >50% decrease in acute tonsillitis episodes at the end of treatment (ie 6 months); “partial response” was defined as a ≤50% decrease in acute tonsillitis episodes at the end of treatment. The immunomodulator used was OM‐85

Microbial‐derived preparations seem to offer an attractive alternative approach in prevention of tonsillitis. Firstly, in a retrospective, observational study of 131 children aged 1‐15 years with recurrent acute tonsillitis, 76% treated with a commercially available bacterial lysate (OM‐85) had a decrease in the frequency of acute tonsillitis episodes after 6 months (Figure 1).^57^ Most (67%) had a greater than 50% decrease in the number of episodes, and none required a tonsillectomy in the subsequent long‐term follow‐up period. Secondly, in a 90‐day trial with the oral probiotic *Streptococcus salivarius* K12, all 30 children who completed the study had a significant reduction in streptococcal pharyngeal infection episodes (more than 90%) compared with the previous year, as well as a significant decrease in the incidence (80%) of oral viral infections^58^; no difference was observed in the control group. Similar results were observed in an earlier study.^59^ Finally, in a study of 160 children aged 5‐14 years with pharyngotonsillitis, ribosomal immunotherapy led to a significant improvement in both specific immunity and non‐specific immunity and may therefore be effective in the prophylaxis of recurrent pharyngotonsillitis.^60^

## 5 OTITIS MEDIA

### 5.1 Epidemiology

Acute OM (AOM) is a viral or bacterial infection of the middle ear and is the most common childhood infection for which antibiotics are prescribed in the USA.^12^, ^61^ Recurrent AOM is typically defined as at least three episodes in a 6‐month period, or four or more episodes in a 12‐month period including at least one episode in the preceding 6 months.^62^ There are a number of risk factors for AOM recurrence, including the winter season, male gender and passive smoking.^62^ Approximately 50% of children aged less than 2 years treated for AOM experience a recurrence within 6 months.^62^ Symptoms that last for more than 10 days may also predict recurrence.^63^

### 5.2 Treatment of acute and recurrent episodes

The treatment approach in AOM depends on factors such as patient age and the severity of signs and symptoms.^62^ Non‐invasive treatment interventions include no treatment/ watchful waiting (but predisposing conditions such as immunodeficiency, anatomic malformations, cystic fibrosis and ciliary dyskinesia need to be excluded); greater patient/parent education, such as highlighting the importance of avoiding passive smoking, as this is a significant factor impairing mucosal immunity; and breastfeeding for children aged less than 1 year.^64^ More invasive interventions, such as the insertion of tympanostomy tubes, may be required for children with recurrent AOM and one or more of the following: aged less than 2 years; underlying medical conditions that predispose the patient to recurrence; or comorbid conditions associated with developmental or language delays. However, there is generally a lack of consensus regarding the role of surgery in AOM.^65^ The parents of children with recurrent AOM are usually anxious about pursuing a surgical treatment option and often seek complementary remedies.^66^

### 5.3 Prevention of recurrent episodes and role of immunomodulation

A key recommendation from the guidelines of the American Academy of Pediatrics (AAP) is that “clinicians should NOT prescribe prophylactic antibiotics to reduce the frequency of episodes of AOM in children with recurrent AOM.”^62^ Pneumococcal and influenza vaccines, given according to the schedule recommended in the AAP guidelines, are recommended.^62^ Indeed, a recent analysis found that the epidemiology of AOM has changed substantially over the past 30 years, highlighted by a decrease in the number of AOM episodes and the number of otitis‐prone children. The authors concluded that this epidemiological shift is associated with the introduction of pneumococcal conjugate vaccines (PCVs) and also due to the development of more stringent diagnostic criteria.^67^ Stopping smoking and banning smoking in public places significantly decreased the rate of children's emergency department visits for middle ear infections and URTIs by 6‐9%, according to a study from Massachusetts, USA.^68^ Probiotics have been shown to be ineffective in patients with AOM.^69^ There may be a role for the use of vitamin D, since levels have been shown to be significantly lower in children with AOM compared with controls.^70^, ^71^ There is also evidence that the incidence of AOM in children can be reduced using immunity‐targeted microbial preparations. For example, the frequency of AOM was assessed as a secondary endpoint in two clinical studies of the bacterial lysate OM‐85 involving children with recurrent acute RTIs. Bacterial lysate treatment reduced the total number of OM episodes by 68% at 6 months (n = 25 vs 8, respectively)^72^ and by 79% at 12 months (n = 14 vs 3, respectively)^73^ compared with placebo. Of note, a randomised controlled Italian study of OM‐85 for the prevention of URTIs (with AOM as a study outcome) in children with risk factors for URTIs and a history of recurrence is planned (EudraCT: 2016‐002705‐19). Ribosomal bacterial immunotherapy has also demonstrated efficacy in children with AOM. In a study of 84 patients aged 4‐14 years, ribosomal immunotherapy led to a significantly improved outcome versus placebo on the incidence of fever, as well as the frequency and duration of infectious episodes.^74^ Ribosomal immunotherapy led to significant improvements in clinical score, immunological parameters, parents' assessment of the patient's symptoms and hearing tests, compared with placebo, in a study of 72 patients aged 6‐14 years.^75^ In addition, a meta‐analysis of 19 randomised, double‐blind studies, including 1215 children, demonstrated a significantly lower need for surgical procedures with ribosomal immunotherapy (1% vs 7% with placebo) in patients with recurrent AOM.^76^

## 6 RECURRENT ACUTE AND CHRONIC RHINOSINUSITIS

### 6.1 Epidemiology

The EPOS group defined acute rhinosinusitis (ARS) as “the sudden onset of two or more symptoms that last for <12 weeks.”^32^ However, in older classifications the term “subacute sinusitis” was proposed, which represents a temporal progression of symptoms for 4‐12 weeks (contemporarily defined as “post‐viral ARS”). Although chronic rhinosinusitis (CRS) is defined as persistence of sinus inflammation for at least 12 weeks, the EPOS group did not feel a separate term to describe patients with prolonged ARS was necessary, and that “exacerbation of CRS” was more appropriate. In Europe, 0.5‐5% of children with URTIs progress to post‐viral ARS^77^, ^78^ and many experience recurrences (point prevalence of 0.035%).^79^ Estimates of CRS prevalence vary significantly worldwide, partly related to differences in the diagnostic criteria used (eg symptom‐based diagnosis vs inclusion of objective rhinoscopy or imaging findings). In the USA, the prevalence of CRS ranges from 2% to 16%, while in various EU countries, it is 7‐27% (average 11%).^77^ Estimates from South America and the Caribbean are in a similar range.^80^, ^81^

Differentiation between recurrent ARS and CRS is difficult, but relies on the complete resolution of symptoms between episodes.^82^ Some patients have recurrent episodes of ARS and may represent a distinct clinical phenotype^83^; these patients should be assessed for underlying risk factors such as allergy, immunodeficiency, cystic fibrosis and anatomical abnormalities,^84^ with consideration of imaging or endoscopic evaluation. As the nasal/paranasal mucosa is the first interface with inhaled toxins and pollutants, environmental factors are thought to be an important cause of transition from ARS to CRS, as well as the trigger for symptom exacerbation in CRS.^85^ For example, there is a significant association between passive smoking and sinusitis.^86^

### 6.2 Treatment of acute and recurrent episodes

Careful analysis of the underlying defect should be performed, for example anatomical abnormalities and immunodeficiency.^87^, ^88^ The prophylactic treatment of recurrent episodes is almost the same as prevention of exacerbation of CRS, that is avoidance of smoking and air pollution, and handwashing to prevent infections. However, in our opinion, the treatment modalities for CRS in children proposed by the Pan American Association in 2011, the EPOS group in 2012 and an International Consensus Statement in 2016, need some refinement, since some important studies have only been published recently while others were overlooked. Firstly, according to a recent Cochrane meta‐analysis evaluating intranasal steroids for CRS in children, there was no effect on disease severity, although symptomatic improvement (nasal blockage, rhinorrhoea, loss of sense of smell and facial pain) was observed.^89^ Based on these observations, we recommend intranasal steroids as a category Ia/A option for children with recurrent ARS and/or CRS.^89^ Secondly, in a randomised, double‐blind study in 51 children, bacterial lysate OM‐85 (recommended as a Ia/A option by EPOS for adults with CRS only, as studies in children were not comprehensively evaluated) not only improved CRS symptoms (nasal discharge and obstruction) and reduced the frequency of CRS exacerbations, but they also decreased the number of days with antibiotic use/month, providing long‐term prophylaxis.^90^ Moreover, this study found that the duration of acute episodes was shorter with bacterial lysates than placebo, suggesting an additional value as a co‐medication for the treatment of CRS.^90^ These observations were supported by two other randomised, double‐blind studies in children (aged 1‐9 years) with recurrent ARS,^91^, ^92^ highlighting the clinical value of non‐specific, immunomodulatory approaches. Table 2 summarises the evidence and recommendations for the management of children with recurrent ARS/exacerbations of CRS as proposed by EPOS, including our own modifications.^32^ A recent international consensus statement also provided evidence‐based recommendations for the treatment of rhinosinusitis.^82^

**Table 2:** Treatment options and recommendations for children with recurrent ARS/CRS exacerbations (adapted from EPOS,^32^ with our own modifications^90^, ^91^, ^92^)

Therapy	Level	Grade of recommendation
Nasal saline irrigation	Ia	A
Topical steroids	Ia	A
Bacterial lysates (OM‐85)	Ib	A
PPI/GERD therapy	Low level evidence or no data	
Topical antimycotics	Low level evidence or no data	
Oral steroids	Low level evidence or no data	
Probiotics	Low level evidence or no data	
Short‐term antibiotics (<4 wk)	Low level evidence or no data	
Intravenous antibiotics	Low level evidence or no data	
Oral long‐term antibiotics	Low level evidence or no data	
Decongestants	Low level evidence or no data	
Mucolytics	Low level evidence or no data	
Oral/topical decongestants	Low level evidence or no data	
Allergen avoidance	Low level evidence or no data	
Allergen immunotherapy	Low level evidence or no data	
Systemic antimycotics	Low level evidence or no data	

Roman numerals indicate evidence levels; capital letters indicate recommendation grades. ARS, acute rhinosinusitis; CRS, chronic rhinosinusitis; EPOS, European Position; GERD, gastroesophageal reflux disease; PPI, proton‐pump inhibitor.

### 6.3 Prevention of recurrent episodes and role of immunomodulation

Immunisation leads to an increase in the host's resistance capabilities and a decrease in the incidence of acute respiratory disease.^93^ Vaccination against pneumococci with the 7‐valent pneumococcal vaccine (PCV7) resulted in a significant shift in the causative pathogens of acute maxillary sinusitis in children; the frequency of *Streptococcus pneumoniae* decreased by 18%, but the proportion of *Haemophilus influenzae* increased by 8%.^94^ Although there is no strong evidence of a change in the incidence of acute bacterial rhinosinusitis after widespread use of PCV, the *H influenzae* type b vaccine has had a positive effect.^95^ Another group of immune‐active agents, bacterial‐derived immunomodulators, has been shown to reduce the number of seasonal URTIs^19^ and decrease the frequency and intensity of ARS episodes or CRS exacerbations.^96^ Based on evidence in adults with CRS without nasal polys,^97^ the EPOS paper and the Pan American Association of Otorhinolaryngology and Head and Neck Surgery guidelines recommend a bacterial lysate (OM‐85), although only in adults.^31^, ^32^ Nevertheless, there is substantial evidence supporting the value of bacterial lysates to reduce the risk for acute RTIs in children, as evidenced by a Cochrane review,^19^ a recent large meta‐analysis of almost 5000 paediatric patients^25^ and clinical studies highlighting the prevention of exacerbations of CRS or recurrent ARS.^90^, ^91^, ^92^ Supported by these findings, several consensus papers and guidelines have noted that OM‐85 could play a role in the prevention of recurrent rhinosinusitis in children.^33^, ^34^, ^98^

Seasonal URTIs are the most common cause of recurrent ARS/exacerbation of CRS. In a Cochrane review evaluating zinc and the common cold, which included 18 randomised controlled trials and 1781 children and adults, it was concluded that zinc could shorten episode duration in children and also be used as a preventative measure^99^; further research should focus on the effect of zinc in patients at a greater risk of developing complications after a common cold. A recent meta‐analysis of 25 randomised controlled trials (including 11 321 children and adults) found that vitamin D supplementation significantly reduced the risk of acute RTIs, with the available evidence assessed as being high quality.^100^ Although a large number of randomised controlled trials have been performed assessing Echinacea and the common cold,^101^ the weakness of trial methods and differences in interventions make it difficult to draw conclusions about its effectiveness in children. Similarly, despite a large number of studies and wide variety of available data, according to a systematic review, evidence supporting the use of vitamin C supplementation and the common cold is limited.^102^

## 7 DISCUSSION

### 7.1 Summary of main findings

Although antimicrobials retain an important role in medicine, their use is becoming less acceptable in modern society, particularly for certain conditions or when administered as a preventative measure. The rapid emergence of resistant bacteria is jeopardising the efficacy of antibiotics; indeed, decreasing the use of antibiotics is considered a top priority for healthcare authorities around the world to avoid the consequent effects of overuse such as an increase in antibiotic resistance and mucosa microbiome impairment.^103^ As such, new treatments and preventative modalities for respiratory infections are expected and welcomed. Modulation of the human immune systems is becoming increasingly relevant, not only in general infectious diseases, allergy or gastroenterology, but also in disciplines such as oncology and ENT‐related disorders. In this review, we have discussed the clinical evidence that supports the use of selected immunomodulatory strategies in children with specific ENT conditions.

### 7.2 Implications for clinical practice

However, there remain some barriers to the widespread use of these therapies. For example, the availability of data on specific ENT infections remain sparse, although evidence around the prevention of general RTIs is more robust. Ideally, new studies will be performed in specific ENT infections in children, in order to increase the evidence base and support treatment recommendations. In addition, according to the available meta‐analysis, many of the commercially available products demonstrate a moderate effect.

### 7.3 Future directions

As such, it is important that we generate high‐quality research data on the use of immunomodulatory strategies in patients with specific URTIs and other upper respiratory tract diseases.

## CONFLICT OF INTEREST

WF has been a speaker for Sandoz, Vifor Pharma, Pierre Fabre and GSK. RM, ASV and KR declare no conflict of interest. JLMB has been a speaker for MSD, Carnot, Grünenthal and has participated as an advisor for MSD and Vifor Pharma.

## References

[1] Jain N , Lodha R , Kabra SK . Upper respiratory tract infections. Indian J Pediatr. 2001;68:1135‐1138. 11838568 10.1007/BF02722930 PMC7091368

[2] Grief SN . Upper respiratory infections. Prim Care. 2013;40:757‐770. 23958368 10.1016/j.pop.2013.06.004 PMC7127764

[3] Sly PD , Holt PG . Role of innate immunity in the development of allergy and asthma. Curr Opin Allergy Clin Immunol. 2011;11:127‐131. 21325945 10.1097/ACI.0b013e32834487c6

[4] Jesenak M , Ciljakova M , Rennerova Z , et al. Recurrent respiratory infections in children – definition, diagnostic approach, treatment and prevention, Bronchitis, Ignacio Martin-Loeches, IntechOpen. https://www.intechopen.com/books/bronchitis/recurrent-respiratoryinfections-in-children-definition-diagnostic-approach-treatment-and-prevention. Accessed October 8, 2018.

[5] Feleszko W , Ruszczynski M , Zalewski BM . Non‐specific immune stimulation in respiratory tract infections. Separating the wheat from the chaff. Paediatr Respir Rev. 2014;15:200‐206. 24275566 10.1016/j.prrv.2013.10.006

[6] Jang AS , Jun YJ , Park MK . Effects of air pollutants on upper airway disease. Curr Opin Allergy Clin Immunol. 2016;16:13‐17. 26658014 10.1097/ACI.0000000000000235

[7] Campbell H . Acute respiratory infection: a global challenge. Arch Dis Child. 1995;73:281‐283. 7492188 10.1136/adc.73.4.281 PMC1511346

[8] Meissner HC . Reducing the impact of viral respiratory infections in children. Pediatr Clin North Am. 2005;52:695‐710. 15925658 10.1016/j.pcl.2005.02.010 PMC7118949

[9] De Sutter A , van Driel ML , Kumar AA , Lesslar O , Skrt A . Oral antihistamine‐decongestant‐analgesic combinations for the common cold. Cochrane Database Syst Rev. 2012;15(2):CD004976. 10.1002/14651858.CD004976.pub3 22336807

[10] Bolser DC . Cough suppressant and pharmacologic protussive therapy: ACCP evidence‐based clinical practice guidelines. Chest. 2006;129:238S–249S. 16428717 10.1378/chest.129.1_suppl.238S PMC3127247

[11] Irwin RS , Baumann MH , Bolser DC , et al. Diagnosis and management of cough executive summary: ACCP evidence‐based clinical practice guidelines. Chest. 2006;129:1S–23S. 16428686 10.1378/chest.129.1_suppl.1S PMC3345522

[12] Fleming‐Dutra KE , Hersh AL , Shapiro DJ , et al. Prevalence of Inappropriate Antibiotic prescriptions among US ambulatory care visits, 2010–2011. JAMA. 2016;315:1864‐1873. 27139059 10.1001/jama.2016.4151

[13] European Centre for Disease Prevention and Control (2016) . Antimicrobial resistance. http://ecdc.europa.eu/en/healthtopics/antimicrobial-resistance-and-consumption/antimicrobial_resistance/Pages/index.aspx. Accessed November 1, 2016.

[14] Carlsen KH , Carlsen KC . Respiratory effects of tobacco smoking on infants and young children. Paediatr Respir Rev. 2008;9:11‐19. 18280975 10.1016/j.prrv.2007.11.007

[15] Arcavi L , Benowitz NL . Cigarette smoking and infection. Arch Intern Med. 2004;164:2206‐2216. 15534156 10.1001/archinte.164.20.2206

[16] Baugh RF , Archer SM , Mitchell RB , et al. Clinical practice guideline: tonsillectomy in children. Otolaryngol Head Neck Surg. 2011;144(1 Suppl):S1‐S30. 21493257 10.1177/0194599810389949

[17] Schaad UB , Principi N . The management of recurrent respiratory tract infections in children. Eur Infect Dis. 2012;6:111‐115.

[18] Schaad UB , Esposito S , Razi CH . Diagnosis and management of recurrent respiratory tract infections in children: a practical guide. Arch Pediatr Infect Dis. 2016;4:e31039.

[19] Del‐Rio‐Navarro BE , Espinosa RF , Flenady V , Sienra‐Monge JJ . Immunostimulants for preventing respiratory tract infection in children (Review). Evid‐Based Child Health. 2012;7:629‐717. 10.1002/14651858.CD004974.pub2 PMC13178731 17054227

[20] Alecsandru D , Valor L , Sánchez‐Ramón S , et al. Sublingual therapeutic immunization with a polyvalent bacterial preparation in patients with recurrent respiratory infections: immunomodulatory effect on antigen‐specific memory CD4+ T cells and impact on clinical outcome. Clin Exp Immunol. 2011;164:100‐107. 21391984 10.1111/j.1365-2249.2011.04320.x PMC3074222

[21] Kearney SC , Dziekiewicz M , Feleszko W . Immunoregulatory and immunostimulatory responses of bacterial lysates in respiratory infections and asthma. Ann Allergy Asthma Immunol. 2015;114:364‐369. 25752734 10.1016/j.anai.2015.02.008

[22] Bousquet J , Fiocchi A . Prevention of recurrent respiratory tract infections in children using a ribosomal immunotherapeutic agent: a clinical review. Paediatr Drugs. 2006;8:235‐243. 16898854 10.2165/00148581-200608040-00003

[23] Fiocchi A , Omboni S , Mora R , et al. Efficacy and safety of ribosome‐component immune modulator for preventing recurrent respiratory infections in socialized children. Allergy Asthma Proc. 2012;33:197‐204. 22525398 10.2500/aap.2012.33.3516

[24] Del‐Rio‐Navarro BE , Espinosa RF , Flenady V , Sienra‐Monge JJ . Immunostimulants for preventing respiratory tract infection in children. Cochrane Database Syst Rev. 2006;(4):CD004974. 17054227 10.1002/14651858.CD004974.pub2 PMC13178731

[25] Yin J , Xu B , Zeng X , Shen K . Broncho‐Vaxom in pediatric recurrent respiratory tract infections: A systematic review and meta‐analysis. Int Immunopharmacol. 2018;54:198‐209. 29154122 10.1016/j.intimp.2017.10.032

[26] Parola C , Salogni L , Vaira X , et al. Selective activation of human dendritic cells by OM‐85 through a NF‐kB and MAPK dependent pathway. PLoS ONE. 2013;8:e82867. 24386121 10.1371/journal.pone.0082867 PMC3875422

[27] Pasquali C , Salami O , Taneja M , et al. Enhanced mucosal antibody production and protection against respiratory infections following an orally administered bacterial extract. Front Med. 2014;1:41. 10.3389/fmed.2014.00041 PMC4292070 25593914

[28] Dang AT , Pasquali C , Ludigs K , Guarda G . OM‐85 is an immunomodulator of interferon‐beta production and inflammasome activity. Sci Rep. 2017;7:43844. 28262817 10.1038/srep43844 PMC5338315

[29] Koatz AM , Coe NA , Ciceran A , Alter AJ . Clinical and immunological benefits of OM‐85 bacterial lysate in patients with allergic rhinitis, asthma, and COPD and recurrent respiratory infections. Lung. 2016;194:687‐697. 27117798 10.1007/s00408-016-9880-5 PMC7087659

[30] Esposito S , Soto‐Martinez ME , Feleszko W , et al. Nonspecific immunomodulators for recurrent respiratory tract infections, wheezing and asthma in children: a systematic review of mechanistic and clinical evidence. Curr Opin Allergy Clin Immunol. 2018;18:198‐209. 29561355 10.1097/ACI.0000000000000433 PMC6037280

[31] Pan American Association of Otorhinolaryngology and Head and Neck Surgery (2011) . Pan American Clinical Practice Guideline for Medical Management of Acute and Chronic Rhinosinusitis. https://www.researchposters.com/Posters/AAOHNSF/aao2012/SP512.pdf. Accessed October 8, 2018.

[32] Fokkens WJ , Lund VJ , Mullol J , et al. EPOS 2012: European position paper on rhinosinusitis and nasal polyps 2012. A summary for otorhinolaryngologists. Rhinology. 2012;50:502‐12. 10.4193/Rhino12.000 22469599

[33] ENT Expert Committee . Expert consensus on the diagnosis, treatment and management of recurrent upper respiratory tract infection in children. Chin J Prac Pediatr. 2017;32:721‐725.

[34] Vietnam Respiratory Association & Vietnam ENT Association . Guidelines for Diagnosis and Treatment of Respiratory Tract Infections in Children. Ha Noi, Vietnam: Nhà xuãt bán Y hoc; 2017.

[35] Defendi GL . Croup. http://emedicine.medscape.com/article/962972-overview. Accessed November 1, 2016.

[36] Worrall G . Croup. Can Fam Physician. 2008;54:573‐574. 18411388 PMC2294095

[37] Woods CR . Patient education: Croup in infants and children (Beyond the Basics). https://www.uptodate.com/contents/croup-in-infants-and-children-beyond-the-basics#H19. Accessed October 8, 2018.

[38] Russell KF , Wiebe N , Saenz A , et al. Glucocorticoids for croup. Cochrane Database Syst Rev. 2004;(1):CD001955. 14973975 10.1002/14651858.CD001955.pub2

[39] Rosychuk RJ , Klassen TP , Metes D , Voaklander DC , Senthilselvan A , Rowe BH . Croup presentations to emergency departments in Alberta, Canada: a large population‐based study. Pediatr Pulmonol. 2010;45:83‐91. 19953656 10.1002/ppul.21162

[40] National Health Service (2014) . Croup – Treatment. http://www.nhs.uk/Conditions/Croup/Pages/Treatment.aspx. Accessed October 8, 2018.

[41] New South Wales Health 2010 . Infants and children: acute management of croup. https://www1.health.nsw.gov.au/pds/ActivePDSDocuments/PD2010_053.pdf. Accessed October 8, 2018

[42] Hryniewicz W . Rekomendacje postepowania w pozaszpitalnych zakazeniach ukladu oddechowego. 2016 http://www.antybiotyki.edu.pl/pdf/Rekomendacje2016.pdf. Accessed October 8, 2018.

[43] Tapiainen T , Aittoniemi J , Immonen J , et al. Finnish guidelines for the treatment of laryngitis, wheezing bronchitis and bronchiolitis in children. Acta Paediatr. 2016;105:44‐49. 26295564 10.1111/apa.13162

[44] Bartlett EJ , Amaro‐Carambot E , Surman SR , et al. Human parainfluenza virus type I (HPIV1) vaccine candidates designed by reverse genetics are attenuated and efficacious in African green monkeys. Vaccine. 2005;23:4631‐4646. 15951066 10.1016/j.vaccine.2005.04.035

[45] Chin J , Magoffin RL , Shearer LA , et al. Field evaluation of a respiratory syncytial virus vaccine and a trivalent parainfluenza virus vaccine in a pediatric population. Am J Epidemiol. 1969;89:449‐463. 4305200 10.1093/oxfordjournals.aje.a120957

[46] Fulginiti VA , Eller JJ , Sieber OF , et al. Respiratory virus immunization. I. A field trial of two inactivated respiratory virus vaccines; an aqueous trivalent parainfluenza virus vaccine and an alum‐precipitated respiratory syncytial virus vaccine. Am J Epidemiol. 1969;89:435‐448. 4305199 10.1093/oxfordjournals.aje.a120956

[47] Karron RA , San MJ , Thumar B , Schaap‐Nutt A . Evaluation of a live‐attenuated human parainfluenza type 1 vaccine in adults and children. J Pediatr Infect Dis Soc. 2015;4:143‐146. 10.1093/jpids/piu104 PMC4681387 26582883

[48] Karron RA , Buchholz UJ , Collins PL . Live‐attenuated respiratory syncytial virus vaccines. Curr Top Microbiol Immunol. 2013;372:259‐284. 24362694 10.1007/978-3-642-38919-1_13 PMC4794267

[49] Windfuhr JP , Toepfner N , Steffen G , Waldfahrer F , Berner R . Clinical practice guideline: tonsillitis I. Diagnostics and nonsurgical management. Eur Arch Otorhinolaryngol. 2016;273:973‐987. 26755048 10.1007/s00405-015-3872-6 PMC7087627

[50] Scottish Intercollegiate Guidelines Network (2010) . Management of sore throat and indications for tonsillectomy: A national clinical guideline. https://www.sign.ac.uk/sign-117-management-of-sore-throat-and-indications-for-tonsillectomy.html. Accessed October 8, 2018.

[51] National Center for Health Statistics (2016) . National ambulatory medical care survey: 1998 summary. http://www.cdc.gov/nchs/. Accessed August 1, 2016.

[52] Kaplan EL , Top Jr FH , Dudding BA , Wannamaker LW . Diagnosis of streptococcal pharyngitis: differentiation of active infection from the carrier state in the symptomatic child. J Infect Dis. 1971;123:490‐501. 5115179 10.1093/infdis/123.5.490

[53] Komaroff AL , Pass TM , Aronson MD , et al. The prediction of streptococcal pharyngitis in adults. J Gen Intern Med. 1986;1:502‐7. 10.1007/BF02596317 3534166

[54] Schroeder BM . Diagnosis and management of group A streptococcal pharyngitis. Am Fam Physician. 2003;67:880‐884. 12613739

[55] American Academy of Otolaryngology‐Head and Neck Surgery (2017) . Tonsillitis. http://www.entnet.org/content/tonsillitis. Accessed October 8, 2018.

[56] Van Brusselen D , Vlieghe E , Schelstraete P , et al. Streptococcal pharyngitis in children: to treat or not to treat? Eur J Pediatr. 2014;173:1275‐1283. 25113742 10.1007/s00431-014-2395-2

[57] Bitar MA , Saade R . The role of OM‐85 BV (Broncho‐Vaxom) in preventing recurrent acute tonsillitis in children. Int J Pediatr Otorhinolaryngol. 2013;77:670‐673. 23380631 10.1016/j.ijporl.2013.01.009

[58] Di Pierro F , Colombo M , Zanvit A , et al. Use of Streptococcus salivarius K12 in the prevention of streptococcal and viral pharyngotonsillitis in children. Drug Healthc Patient Saf. 2014;6:15‐20. 24600248 10.2147/DHPS.S59665 PMC3928062

[59] Di Pierro F , Adami T , Rapacioli G , et al. Clinical evaluation of the oral probiotic Streptococcus salivarius K12 in the prevention of recurrent pharyngitis and/or tonsillitis caused by Streptococcus pyogenes in adults. Expert Opin Biol Ther. 2013;13:339‐343. 23286823 10.1517/14712598.2013.758711

[60] Mora R , Dellepiane M , Crippa B , Salami A . Ribosomal therapy in the prophylaxis of recurrent pharyngotonsillitis in children. Int J Pediatr Otorhinolaryngol. 2007;71:257‐261. 17126918 10.1016/j.ijporl.2006.10.007

[61] McCaig LF , Besser RE , Hughes JM . Trends in antimicrobial prescribing rates for children and adolescents. JAMA. 2002;287:3096‐3102. 12069672 10.1001/jama.287.23.3096

[62] Lieberthal AS , Carroll AE , Chonmaitree T , et al. The diagnosis and management of acute otitis media. Pediatrics. 2013;131:e964‐e999. 23439909 10.1542/peds.2012-3488

[63] Damoiseaux RA , Rovers MM , Van Balen F , Hoes AW , de Melker RA . Long‐term prognosis of acute otitis media in infancy: determinants of recurrent acute otitis media and persistent middle ear effusion. Fam Pract. 2006;23:40‐45. 16107490 10.1093/fampra/cmi083

[64] Klein JO , Pelton S . Patient education: ear infections (otitis media) in children (Beyond the Basics). 2016 http://www.uptodate.com/contents/ear-infections-otitis-media-in-children-beyond-the-basics. Accessed October 8, 2018.

[65] Schilder A , Marom T , Bhutta MF , et al. Panel 7: otitis Media: treatment and complications. Otolaryngol. Head Neck Surg. 2017;156:S88‐S105. 28372534 10.1177/0194599816633697

[66] Marom T , Marchisio P , Tamir SO , Torretta S , Gavriel H , Esposito S . Complementary and alternative medicine treatment options for otitis media: A systematic review. Medicine. 2016;95:e2695. 26871802 10.1097/MD.0000000000002695 PMC4753897

[67] Kaur R , Morris M , Pichichero ME . Epidemiology of acute otitis media in the postpneumococcal conjugate vaccine era. Pediatrics. 2017;140(3):e20170181. 28784702 10.1542/peds.2017-0181 PMC5574724

[68] Hawkins SS , Hristakeva S , Gottlieb M , Baum CF . Reduction in emergency department visits for children's asthma, ear infections, and respiratory infections after the introduction of state smoke‐free legislation. Prev Med. 2016;89:278‐285. 27283094 10.1016/j.ypmed.2016.06.005 PMC8323994

[69] Cohen R , Martin E , de La Rocque F , et al. Probiotics and prebiotics in preventing episodes of acute otitis media in high‐risk children: a randomized, double‐blind, placebo‐controlled study. Pediatr Infect Dis J. 2013;32:810‐814. 23429555 10.1097/INF.0b013e31828df4f3

[70] Cayir A , Turan MI , Ozkan O , Cayir Y . Vitamin D levels in children diagnosed with acute otitis media. J Pak Med Assoc. 2014;64:1274‐1277. 25831645

[71] Li HB , Tai XH , Sang YH , et al. Association between vitamin D and development of otitis media: A PRISMA‐compliant meta‐analysis and systematic review. Medicine. 2016;95:e4739. 27749530 10.1097/MD.0000000000004739 PMC5059032

[72] Jara‐Perez JV , Berber A . Primary prevention of acute respiratory tract infections in children using a bacterial immunostimulant: a double‐masked, placebo‐controlled clinical trial. Clin Ther. 2000;22:748‐759. 10929921 10.1016/S0149-2918(00)90008-0

[73] Gutierrez‐Tarango MD , Berber A . Safety and efficacy of two courses of OM‐85 BV in the prevention of respiratory tract infections in children during 12 months. Chest. 2001;119:1742‐1748. 11399700 10.1378/chest.119.6.1742

[74] Mora R , Barbieri M , Passali GC , et al. A preventive measure for otitis media in children with upper respiratory tract infections. Int J Pediatr Otorhinolaryngol. 2002;63:111‐118. 11955602 10.1016/s0165-5876(01)00649-8

[75] Mora R , Ralli G , Passali FM , et al. Short ribosomal prophylaxis in the prevention of clinical recurrences of chronic otitis media in children. Int J Pediatr Otorhinolaryngol. 2004;68:83‐89. 14687691 10.1016/j.ijporl.2003.09.008

[76] Bellanti J , Olivieri D , Serrano E . Ribosomal immunostimulation: assessment of studies evaluating its clinical relevance in the prevention of upper and lower respiratory tract infections in children and adults. BioDrugs. 2003;17:355‐367. 14498765 10.2165/00063030-200317050-00005

[77] Hastan D , Fokkens WJ , Bachert C , et al. Chronic rhinosinusitis in Europe–an underestimated disease. A GA(2)LEN study. Allergy. 2011;66:1216‐1223. 21605125 10.1111/j.1398-9995.2011.02646.x

[78] Lusk R . Pediatric chronic rhinosinusitis. Curr Opin Otolaryngol Head Neck Surg. 2006;14:393‐396. 17099346 10.1097/MOO.0b013e32801000ed

[79] Bhattacharyya N , Grebner J , Martinson NG . Recurrent acute rhinosinusitis: epidemiology and health care cost burden. Otolaryngol Head Neck Surg. 2012;146:307‐312. 22027867 10.1177/0194599811426089

[80] Ahsan SF , Jumans S , Nunez DA . Chronic rhinosinusitis: a comparative study of disease occurrence in North of Scotland and Southern Caribbean otolaryngology outpatient clinics over a two month period. Scott Med J. 2004;49:130‐133. 15648704 10.1177/003693300404900404

[81] Pilan RR , Pinna FR , Bezerra TF , et al. Prevalence of chronic rhinosinusitis in Sao Paulo. Rhinology. 2012;50:129‐138. 22616073 10.4193/Rhino11.256

[82] Orlandi RR , Kingdom TT , Hwang PH , et al. International consensus statement on allergy and rhinology: Rhinosinusitis. Int Forum Allergy Rhinol. 2016;6:S22‐S209. 26889651 10.1002/alr.21695

[83] Poetker DM , Litvack JR , Mace JC , Smith TL . Recurrent acute rhinosinusitis: presentation and outcomes of sinus surgery. Am J Rhinol. 2008;22:329‐333. 18588769 10.2500/ajr.2008.22.3177

[84] Alkire BC , Bhattacharyya N . An assessment of sinonasal anatomic variants potentially associated with recurrent acute rhinosinusitis. Laryngoscope. 2010;120:631‐634. 20131360 10.1002/lary.20804

[85] Sundaresan AS , Hirsch AG , Storm M , et al. Occupational and environmental risk factors for chronic rhinosinusitis: a systematic review. Int Forum Allergy Rhinol. 2015;5:996‐1003. 26077513 10.1002/alr.21573 PMC4681694

[86] Hur K , Liang J , Lin SY . The role of secondhand smoke in sinusitis: a systematic review. Int Forum Allergy Rhinol. 2014;4:22‐28. 24574074 10.1002/alr.21232

[87] Chiarella SE , Grammer LC . Immune deficiency in chronic rhinosinusitis: screening and treatment. Expert Rev Clin Immunol. 2017;13:117‐123. 27500811 10.1080/1744666X.2016.1216790 PMC5429028

[88] Schwitzguébel A‐P , Jandus P , Lacroix J‐S , Seebach JD , Harr T . Immunoglobulin deficiency in patients with chronic rhinosinusitis: Systematic review of the literature and meta‐analysis. J Allergy Clin Immunol. 2015;136:1523‐1531. 26329513 10.1016/j.jaci.2015.07.016

[89] Chong LY , Head K , Hopkins C , Philpott C , Schilder A , Burton MJ . Intranasal steroids versus placebo or no intervention for chronic rhinosinusitis. Cochrane Database Syst Rev. 2016;4:CD011996. 27115217 10.1002/14651858.CD011996.pub2 PMC9393647

[90] Zagar S , Lofler‐Badzek D . Broncho‐Vaxom in children with rhinosinusitis: a double‐blind clinical trial. ORL J Otorhinolaryngol Relat Spec. 1988;50:397‐404. 3068610 10.1159/000276020

[91] Gomez BD , De la TC , Alvarez A , et al. Safety and efficacy of OM‐85‐BV plus amoxicillin/clavulanate in the treatment of subacute sinusitis and the prevention of recurrent infections in children. Allergol Immunopathol. 1998;26:17‐22. 9585823

[92] Razi CH , Harmanci K , Abaci A , et al. The immunostimulant OM‐85 BV prevents wheezing attacks in preschool children. J Allergy Clin Immunol. 2010;126:763‐769. 20920766 10.1016/j.jaci.2010.07.038

[93] Goodridge HS , Ahmed SS , Curtis N , et al. Harnessing the beneficial heterologous effects of vaccination. Nat Rev Immunol. 2016;16:392‐400. 27157064 10.1038/nri.2016.43 PMC4931283

[94] Brook I , Gober AE . Frequency of recovery of pathogens from the nasopharynx of children with acute maxillary sinusitis before and after the introduction of vaccination with the 7‐valent pneumococcal vaccine. Int J Pediatr Otorhinolaryngol. 2007;71:575‐579. 17267051 10.1016/j.ijporl.2006.10.025

[95] Benninger MS , Manz R . The impact of vaccination on rhinosinusitis and otitis media. Curr Allergy Asthma Rep. 2010;10:411‐418. 20737301 10.1007/s11882-010-0139-6

[96] Chen J , Zhou Y , Nie J , et al. Bacterial lysate for the prevention of chronic rhinosinusitis recurrence in children. J Laryngol Otol. 2017;131:523‐528. 28318451 10.1017/S0022215117000524

[97] Heintz B , Schlenter WW , Kirsten R , Nelson K . Clinical efficacy of Broncho‐Vaxom in adult patients with chronic purulent sinusitis–a multi‐centric, placebo‐controlled, double‐blind study. Int J Clin Pharmacol Ther Toxicol. 1989;27:530‐534. 2693373

[98] Anselmo‐Lima WT , Sakano E , Tamashiro E , et al. Rhinosinusitis: evidence and experience. Braz J Otorhinolaryngol. 2015;81:S1‐S49. 25697512 10.1016/j.bjorl.2015.01.003 PMC10157818

[99] Singh M , Das RR . Zinc for the common cold. Cochrane Database Syst Rev. 2015;(4):CD001364. 25924708 10.1002/14651858.CD001364.pub5 PMC6457799

[100] Martineau AR , Jolliffe DA , Hooper RL , et al. Vitamin D supplementation to prevent acute respiratory tract infections: systematic review and meta‐analysis of individual participant data. BMJ. 2017;356:i6583. 28202713 10.1136/bmj.i6583 PMC5310969

[101] Karsch‐Völk M , Barrett B , Kiefer D , Bauer R , Ardjomand‐Woelkart K , Linde K . Echinacea for preventing and treating the common cold. Cochrane Database Syst Rev. 2014;(2):CD000530. 24554461 10.1002/14651858.CD000530.pub3 PMC4068831

[102] Hemilä H , Chalker E . Vitamin C for preventing and treating the common cold. Cochrane Database Syst Rev. 2013;(1):CD000980. 23440782 10.1002/14651858.CD000980.pub4 PMC8078152

[103] World Health Organization (2015) . Global action plan on antimicrobial resistance. http://apps.who.int/iris/bitstream/10665/193736/1/9789241509763_eng.pdf?ua=1. Accessed October 8, 2018.

